# Reactive oxygen species-responsive polymer drug delivery systems

**DOI:** 10.3389/fbioe.2023.1115603

**Published:** 2023-02-02

**Authors:** Jiaxue Liu, Boyan Jia, Zhibo Li, Wenliang Li

**Affiliations:** ^1^ Jilin Collaborative Innovation Center for Antibody Engineering, Jilin Medical University, Jilin, China; ^2^ Department of Cardiology, The Second Hospital of Jilin University, Changchun, China

**Keywords:** reactive oxygen species (ROS), ROS-responsive, nanoparticle, hydrogel, stimuli-responsive

## Abstract

Applying reactive polymer materials sensitive to biological stimuli has recently attracted extensive research interest. The special physiological effects of reactive oxygen species (ROS) on tumors or inflammation and the application of ROS-responsive polymers as drug-delivery systems in organisms have attracted much attention. ROS is a vital disease signal molecule, and the unique accumulation of ROS-responsive polymers in pathological sites may enable ROS-responsive polymers to deliver payload (such as drugs, ROS-responsive prodrugs, and gene therapy fragments) in a targeted fashion. In this paper, the research progress of ROS-responsive polymers and their application in recent years were summarized and analyzed. The research progress of ROS-responsive polymers was reviewed from the perspective of nanoparticle drug delivery systems, multi-responsive delivery systems, and ROS-responsive hydrogels. It is expected that our work will help understand the future development trends in this field.

## Graphical Abstract

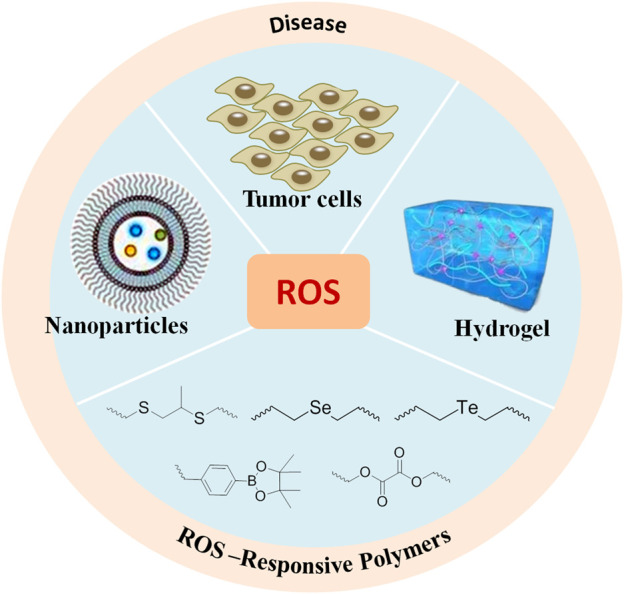

## 1 Introduction

Reactive oxygen species (ROS) are substances in the body or natural environment composed of oxygen and have active properties. They are a class of single-electron reduction products of oxygen in the body, including hydrogen peroxide (H_2_O_2_), superoxide (1O_2_), hydroxyl radical (OH), peroxynitrite (ONOO^−^), and hypochlorite (OCl^−^) (Kearns, 1971; Lissi et al., 1993; Schweitzer and Schmidt, 2003; Gligorovski et al., 2015; Hayyan et al., 2016; Nosaka and Nosaka, 2017). ROS are by-products of cell metabolism, mainly from the mitochondria and plasma membranes. In the body, ROS is formed when electrons escape the respiratory chain and consume oxygen before reaching the terminal oxidase during the transmission process of the inner mitochondrial membrane. O_2_ is reduced to O_2_^-^ or H_2_O_2_, the precursor of most ROS (Chen et al., 2003; Murphy, 2009). Another important source of ROS is NADPHase, which is expressed on the cytoplasmic membrane and generates ROS by plasmic electron transport, which can be present in large numbers in phagocytes or at low levels in various tissues and cells (Robinson, 2008).

As a by-product of metabolism, ROS is a class of molecules with high activity, which plays an essential role in physiological (low ROS) or pathological (high ROS) processes. At low ROS levels, ROS can act as a signal for small molecules involved in maintaining physiological conditions, such as signaling activation of many downstream membrane receptors and maintaining the normal function of cells (Valko et al., 2007; Banerjee et al., 2017; Dan et al., 2021; Dan et al., 2022). While at high ROS levels, the balance between ROS production and the antioxidant systems is lost, resulting in oxidative stress (Di Meo et al., 2016; Huan et al., 2019). This subsequently destroys intracellular biomacromolecules (such as nucleic acids, membrane lipids, and cellular proteins). Oxidative damage to these biomolecules triggers apoptosis and is closely associated with the pathogenesis of aging (Ishii et al., 1998) and many diseases, such as cancer, neurodegenerative diseases, diabetes, and inflammation (Barnham et al., 2004; Fraisl et al., 2009; Trachootham et al., 2009; Schieber and Chandel, 2014; Shi et al., 2022).

ROS overexpression is related to the occurrence and development of disease. We know that it can destroy biological macromolecules and affect gene expression. Additionally, cell proliferation and differentiation are also affected, leading to reduced cell apoptosis, excessive cell proliferation, inflammation, and even tumor formation (Schieber and Chandel, 2014). ROS overexpression and elevation have been observed in many diseases (Barnham et al., 2004; Trachootham et al., 2009), such as tumor tissues. Under physiological conditions, intracellular ROS levels are finely regulated to act as messengers during normal cell signal transduction, cell cycle, gene expression, and homeostasis. Thus, the ROS concentration in normal tissues is always about 20 nmol/L. However, because of the production and accumulation of excess H_2_O_2_ in tumor tissues, the H_2_O_2_ concentration reaches approximately 50–100 nmol/L. In addition, elevated ROS levels are seen in inflammation, other diseases.

The research and application of bioresponsive polymers have been extensive, and the development of pH, heat-sensitive, light-sensitive, and other responsive materials has seen rapid growth. ROS plays a unique signaling role in pathological development and abnormally accumulates at pathological sites. Therefore, it is considered a target or indicator different from the surrounding normal tissue environment at sites with inflammation and tumors. ROS-responsive materials are considered for site-specific delivery of therapeutic and imaging agents or as implanted hydrogels that degrade and release therapeutic agents *in vivo* (Sue et al., 2013). Different ROS-responsive groups can adjust the intracellular or external ROS levels to alleviate inflammation progression, protect genes, and treat diseases. In addition, by taking advantage of the characteristics of high ROS in the tumor microenvironment, drug delivery can be targeted to tumor cells, and the safety of chemotherapy drugs can be significantly improved.

While polymer nanomicelles can effectively deliver therapeutic drugs to disease sites, irritation-responsive nanoparticles are becoming increasingly popular because of their ability to release specific payloads. Stimulus-responsive nanoparticles are usually stable in blood circulation and normal physiological activities. However, once they infiltrate the body, they are activated by tumor microenvironments such as acid or enzymatic upregulation or hypoxia. Nanoparticles are usually expected to be first “inactive” in the body’s circulatory system, “docked” at the tumor site *via* enhanced permeability and retention (EPR) effects, and then “activated” within the tumor’s interstitial space to release drugs or indicators. Therefore, with good biodistribution and pharmacokinetic properties, the rapid diffusion and penetration properties are considered. High ROS physiological levels represent a wide range of tumor physiological environments without tumor specificity. The design of relevant, responsive materials and therapeutics has the potential to overcome the problem of tumor heterogeneity and be used in the diagnosis and treatment of solid tumors (Jing and Bin, 2016). The current methods used for drug delivery are mainly based on “active” and “passive” mechanisms. In passive mode, because the tumor site is highly permeable with abnormal blood vessels at the tumor site (looser capillaries and a damaged lymphatic system), nanoparticles without targeted ligands accumulate in the tumor region, causing EPR effects. In active mode, molecular ligands such as antibodies, peptides, or small molecules that typically bind to specific receptors on tumor cell membranes (and are subsequently internalized by receptor-mediated endocytosis) are added to the surface of particles. In both mechanisms, the arrival of nanomedicine at the tumor site is the first step. The key to treatment is the rapid internalization of drugs, killing of tumor cells, and elimination of inflammation. Recent advances have also focused on developing stimulus-responsive nanoparticles targeting tumors or inflammatory microenvironments (Rong et al., 2017; Meng et al., 2018). Using the microenvironmental characteristics of the tumor or inflammation itself, stimulus-triggered nanoparticles are activated under certain conditions, accelerating drug release at the targeting site, improving cell binding and internalization, or more effectively delivering drugs throughout the tumor volume.

ROS-responsive drug delivery systems are based on the unique redox microenvironment of the tumor or inflammation. Related ROS-responsive platforms have demonstrated their potential for many biomedical applications, such as targeted drug delivery systems for cancer and cell therapy platforms for inflammation-related diseases (Xusheng et al., 2018; Jayachandra et al., 2021). Many ROS-responsive materials are being explored, including thioether, selenium, tellurium, thiokesterone, polysaccharide, amino acrylate, borate, peroxalate, and polyproline. The responsive linker can be selected according to the design purpose of the material, the carrier, and the release characteristics of the drugs. This review is mainly based on the current research and application progress of ROS-responsive polymer materials and is expected to be helpful to the current research.

## 2 ROS-responsive polymers

ROS-responsive materials refer to a class of materials with chemical structures that change under high ROS levels, such as hydrophilic and hydrophobic changes and fractures. This reactive polymer material is highly modifiable and selective. This allows it to release drugs under different ROS levels, provide precise localized treatment, reduce the damage of drugs to normal tissues, and improve the accumulation of drugs in tumors and other sites, thus, improving the therapeutic effect. These materials include sulfur-based, selenium-based, and tellurium-based responsive polymers and phenyl boric acid-based polymer and oxalic acid materials (Shim and Xia, 2013; Cao et al., 2015; Xiao et al., 2015). The common ROS-responsive groups are shown in Table 1.

**TABLE 1:** Common ROS-responsive groups.

ROS-responsive materials	ROS-responsive groups	Responsive concentration of H_2_O_2_ (μmol/L)
Sulfur-based responsive polymers	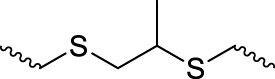	50
Selenium-based responsive polymers	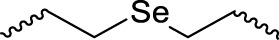	100
Tellurium-based responsive polymers	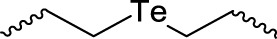	100
Phenyl boric acid-based polymer materials	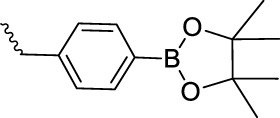	10
Oxalic acid-based polymer materials	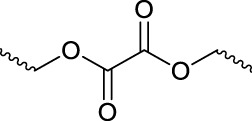	50

### 2.1 Sulfur-based responsive polymers

Sulfur-containing polymers are reductive and can be oxidized in the presence of ROS. Some sulfur-containing responsive structures are summarized in Table 2. These structures can respond to most ROS components, allowing designers to choose according to the material needs. In sulfur-containing structures, the structure of thioether obtained in polymers changes from a hydrophobic to a hydrophilic structure after the oxidation of oxide to the sulfoxide. Additionally, the change in water solubility can realize the responsive release of drugs in the ROS environment. Keto thiols break chemical bonds in the ROS environment, resulting in ketones, thiols, and responsive release drugs.

**TABLE 2:** Chemical structures of the sulfur-based units.

Sulfur-based ROS-responsive units		Known-response
Sulfide/thioether	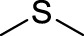	H_2_O_2_, OH^−^, ClO^−^
Bis(alkylthio)alkene/vinyl disulfide	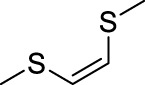	O^−^
Sulfoxide	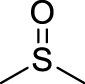	H_2_O_2_, OH^−^, ClO^−^, Organic peroxides
Thioketal	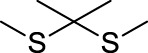	H_2_O_2_, OH^−^, ClO^−^, O^−^, Superoxide
Disulfide	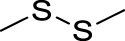	Almost all ROS
Oligosulfide	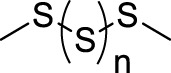	Almost all ROS

Poly (propylenesulfide) (PPS) is a hydrophobic polymer with a thioether structure. The hydrophobicity of PPS can co-load drugs, and the triple-block polymer constructed by PPS and PEG can form a vesicle structure in an aqueous solution. The vesicle structure can be oxidized slowly at low H_2_O_2_ concentrations and rapidly oxidized and disintegrated at high H_2_O_2_ concentrations to release drugs (Napoli et al., 2004). Micelles composed of poly (glycidyl methacrylate)-polyacrylamide sulfide (PGED-PPS) loaded with simvastatin, together with red blood cells (RBCs), constitute intelligent response systems that simultaneously respond to ROS and the stress microenvironment of atherosclerotic plaques. The hydrophobic PPS in the micelles may react with excess ROS to become hydrophilic, which forces the micelles to rupture, resulting in drug release. Most importantly, PPS can also significantly deplete ROS levels, enabling synergistic treatment of atherosclerosis with drugs and materials (Meili et al., 2021).

Ketothiol is ROS-responsive, and its polymers can be loaded with chemotherapeutic drugs. In areas with high levels of ROS, such as inflammation and tumors, ketothiol chemical bonds are broken, and drugs are released for treatment. For example, nanoparticles composed of poly (1, 4-phenylacetone dimethylene thioacetone) (PPADT) and polythioacetone ethyl carbamate (PTKU) encapsulate dexamethasone acetate (Dex) to form polymer nanoparticles (NPs) (PTKNPs@Dex). Thus, a therapeutic nanoplatform with ROS responsiveness was developed to modulate inflammation. Thioketal bonds in the nanoparticles can be cleaved by high levels of ROS at the ALI site. PTKNPs@Dex can accumulate and rapidly release encapsulated payloads at sites of inflammation in the lung, resulting in lower ROS levels, reduced proinflammatory cytokine production, and reduced lung injury and mortality in mice. RNA sequencing (RNA-Seq) analysis showed that the therapeutic effect of NPs was related to the regulation of many immune and inflammatory pathways. These findings provide a newly developed nanoplatform for the effective treatment of ALI/ARDS (Zihe et al., 2022).

The ROS cleavable cationic polymer synthesized by polymerization of oligomeric amine and acrylamide thiokeone cross-linker is a poly (amino thioketal) (PATK). PATK based on ROS-responsive thiokeones is safe, efficient, and targeted for gene delivery to prostate cancer cells. PATK showed a good degradation effect with H_2_O_2_. Therefore, PATK can release complex DNA efficiently, and the DNA/PATK complex has a good gene transfection effect in prostate cancer cells (Min and Younan, 2013).

Xu et al. synthesized MPEG-poly (ester-thioether), an amphiphilic copolymer of MPEG and poly (thioacetone-ester) for the manufacture of ROS polymers for drug delivery. The ROS sensitivity of copolymers was confirmed *in vitro*. MPEG-poly (ester-thioether) nanoparticles exhibited the fastest drug release rate and had the best ROS sensitivity (Xu et al., 2019).

### 2.2 Selenium-based responsive polymers

Selenium (Se) has similar properties to sulfur but also has reducing properties. Because of the higher sensitivity and responsiveness of Se-containing polymers and lower bond energies, the development of Se-containing drug carriers and other biomaterials has received increased attention. Hydrophobic Se groups can be transformed into hydrophilic selenosulfone in a ROS environment, which can depolymerize micelles or nanoparticles, thus resulting in drug release. The Se-Se group will break in the ROS environment and be oxidized to selenic acid, and the corresponding nanoparticles or micelles will release the drug during the depolymerization process.

Zhang et al. investigated polymers containing single Se for ROS drug delivery systems. The PEG-PuSe-PEG triblock copolymer was synthesized, and the mono-sulfide PEG-PuS-PEG was prepared as a control. Both materials showed ROS responsiveness when the drug-loaded micelles prepared from block copolymers were exposed to ROS oxidation. However, the Se-containing micelles had higher response activity, and the release rate of the micelles at the concentration of .1% H_2_O_2_ (v/v) for 10 h, was twice that of the sulfur-containing micelles, reaching more than 70% (Ning et al., 2010a). Polymers with side chains containing selenium also showed responsiveness in the ROS environment and were used as drug carriers to prepare polymer micelles for relevant studies (Peng et al., 2010). Se-containing hyperbranched polymers and their synthesis and application as drug carriers have also received relevant attention (Cao et al., 2013; Xiaofang et al., 2021; Ming et al., 2022). Lin et al. explored the polymerization of Se-containing hyperbranched polymers (Se-HBPs) by disselenium substitution. A series of Se-HBPs with controllable molecular weight and definite structure were obtained by systematically optimizing reaction conditions. The degree of branching (DB) of this type of polymer is almost as high as that of dendritic polymers. Fluorescence, aggregation-induced emission (AIE), and amphipathic Se-HBP can be prepared in a one-pot reaction. Notably, the AIE-terminal Se-HBP is quenched by a combination of AIE and aggregation (ACQ). Amphipathic Se-HBPs can be self-assembled into nanoparticles with a good oxidation response. It is sensitive to .01 wt% H_2_O_2_ oxidation (Xiaofang et al., 2021). Moreover, the Se-Se bond can be oxidized to selenic acid (.01% H_2_O_2_) or reduced to selenol (.01% GSH) by reducing agents in an oxidizing environment because of its unique bi-redox sensitivity. Micelles based on PEG-block-Se-Se-polyetherane-block-PEG triblock copolymers (PEG-b-PuSeSe-b-PEG) showed triggered dissolution in the presence of an oxidant or reducing agent. Zhang and colleagues first developed a triblock copolymer composed of poly (ethylene glycol)-b-polyurethane-b-poly (ethylene glycol) (PEG-PuSeSe-PEG) using the disenene bond. The developed polymers can self-assemble in water to form micelles and release drugs upon stimulation of glutathione (GSH) and H_2_O_2_. Furthermore, other studies showed that these micelles were also responsive to *γ*-radiation, demonstrating their potential application value in combined radiotherapy and chemotherapy (Ning et al., 2010b). Wang et al. developed a photoresponsive nanogel composed of polymer *p* (MAA-Se-Se-MAA), in which indocyanine green (ICG) can generate 1O_2_ under the action of light and then decompose the nanogel formed by the cross-linking polymer on demand. The controlled release of DOX under light (785 nm) and its possible anticancer effect have been demonstrated *in vitro*, which shows great potential for targeted chemotherapy (Ian et al., 2015).

### 2.3 Tellurium-based responsive polymers

Tellurium (Te) is more reductive than sulfur and selenium. Te-containing compounds have higher ROS responsiveness and sensitivity compared with Se-containing compounds. Therefore, studies on Te-containing compounds have attracted increased attention and have been developed as promising biomaterials (Mao et al., 2005; Liu et al., 2009; Thomas et al., 2012; Yu et al., 2013). In the H_2_O_2_ environment, the Te-containing polymer is oxidized from a low to a high state, and the solubility of the material changes to realize drug release. Te-containing compounds are also responsive to radiation signals, which makes it possible to design multistage synergistic drug release systems or combined photodynamic and photothermal treatment platforms.

Xu et al. systematically studied the biological application of Te-containing polymers. They synthesized a Te-containing polyurethane material (PEG-PuTe-PEG) and studied its ROS responsiveness in depth (Cao et al., 2015). Studies have shown that the micelles formed by the self-organization of the Te-containing polyurethane PEG-PuTe-PEG rapidly expand in volume and release drugs in the presence of H_2_O_2_ (.1 mM). Cyclic voltammetry measurements were also used to identify polyurethanes containing Te, which are more sensitive to redox than polymers containing selenium and sulfur. In addition, Te-containing polymers are responsive to ionizing and 2 gy gamma-ray radiation, indicating that the materials have a more comprehensive range of potential applications worth developing and studying. In 2017, PEG-PuTe-PEG was used to prepare a drug-loaded nanoplatform based on the systematic interaction of Te and cisplatin. Indocyanine green (ICG) was added to the system, and the release of cisplatin was controlled by infrared irradiation (Li et al., 2017). The same research group synthesized and prepared hyperbranched Te-containing hyperbranched polymers (HBPTe 1,900). Micelles formed from polymers can respond to low concentrations of ROS (.1 μmol L^−1^ H_2_O_2_). In the ROS response experiment, the particle size of the Te hyperbranched polymer micelles changed significantly in the H_2_O_2_ solution. It was proved that HBPTe 1,900 was a potential ROS-responsive vector. However, the specific drug-loading nanoplatform and timely drug-release mechanism must be further studied (Fang et al., 2015).

### 2.4 Phenyl boric ester-based responsive polymers

Phenyl boric acid and its derivatives are the groups for constructing organic compounds and functional polymers. Phenyl boric acid and phenyl borate ester are oxidized to boric acid by ROS, the boric acid is hydrolyzed to phenol and boric acid, and the chemical bond is broken to allow for drug release. Phenyl borate can respond to ROS at physiological concentrations (Lux et al., 2012). As one of the most sensitive functional groups to ROS, this has become a promising biological material. There are many reports on developing ROS-responsive biomaterials based on aryl borate groups.

Jaeger et al. (2016) reported the preparation and use of fluorescent arylboroester-based polymers for ROS-triggered drug release and ROS detection. The response of nanoparticles assembled from polymers to H_2_O_2_ concentrations was as low as .2 mM. Sun et al. prepared a degradable branched polyethyleneimine containing carbon and boron bonds through a borate bond cross-linking reaction, which was used as a carrier for gene delivery and targeted therapy for breast cancer. The results showed that the prepared BPEI/plasmid DNA nanoparticles had better biocompatibility, ROS cleavage ability, targeting ability, and a higher transfection efficiency compared with BPEI *in vitro* and *in vivo*. *In vitro* experiments showed that the plasmid had an excellent gene-silencing effect, and *in vivo* experiments showed that the material had a good antitumor ability (Ruan et al., 2018). Liu et al. reported amphiphilic block copolymers of borate containing aryl groups (PEO-B-PPBMA, PEO-B-PPBCMA, PEO-B-PNBMA, and PEO-B-PNBCMA). All four amphiphilic block copolymers contain phenylboronate (PB) and naphthylboronate (NB) esters, so they are ROS-responsive. The assembled nanocarriers have ROS-triggered double-layer cross-linking, permeability inversion, enhanced imaging, and drug release behavior (Deng et al., 2016). Shen et al. developed an arylboroester bonded cationic polymer (B PDEAEA) that can undergo charge reversal in response to ROS-triggering and can be used as a gene carrier for gene therapy. Their experiments confirmed that the designed polymer could degrade quickly in 1 mM H_2_O_2_, completing the charge reversal process within 10 h. *In vitro* gene transfection rate experiments showed that the transfection efficiency of the plasmid composed of the material gene was satisfactory, which could reach nine times the gold standard of gene transfection using branch polyethyleneimine (PEI, 25 kDa) (Liu et al., 2016).

### 2.5 Oxalate ester-containing polymers

Oxalate polymers are reactive to ROS, and oxalate is easily oxidized to alcohol and CO_2_ by reacting with the oxidant H_2_O_2_. Nanoparticles composed of oxalate polypolymers can be induced by oxidants to descend and release cargo upon exposure to H_2_O_2_ to achieve drug release. Therefore, oxalate polymers are potential oxidation-responsive nanocarriers, and related materials have been developed and applied. The oxalate ester-containing polymers A (OEPA) prepared by Lee et al. (2007) can be coated with fluorescent pigments to make fluorescent dye-loaded nanoparticles. *In vivo* imaging experiments show that the OEPA-loaded fluorescent dye nanoparticles can be used for *in vivo* imaging. In addition, the lowest concentration of H_2_O_2_ that can be detected by the fluorescent dye nanoparticles prepared by OEPA is 250 nmol L^−1^. This group also synthesized oxalate ester-containing polymers B (OEPB) and prepared the loaded fluorescent dye micelles for cellular H_2_O_2_ imaging, which could detect approximately 50 nmol L^−1^ H_2_O_2_ (Lee et al., 2008).

Lee et al. reported a new type of nanotherapeutic agent. The nanoparticles assembled based on the antioxidant polyoxalic vanillic aloxalate (PVO) triggered by H_2_O_2_ to produce CO_2_. When H_2_O_2_ was present, the diameter of PVO nanoparticles increased significantly and expanded, and bubbles were formed. The nanoparticle expressed the effect of ultrasound imaging and concurrent treatment of hepatic ischemia/reperfusion (I/R). H_2_O_2_ can trigger the oxidation of peroxalate, and the PVO nanoparticles generate CO_2_ and release vanillin, thus playing antioxidant and anti-inflammatory roles. Studies have shown that intravenously administered PVO nanoparticles can significantly enhance the ultrasound signal at the site of hepatic ischemia/reperfusion (I/R) injury and effectively inhibit liver injury by inhibiting inflammation and apoptosis. This H_2_O_2_-responsive PVO is the first platform to generate bubbles for use as an ultrasound contrast agent and to exert therapeutic effects (Kang et al., 2016).

Huang et al. synthesized poly (vanillanol-co-oxalate) (PVAX) polymer for the inclusion of curcumin (CUR). PVAX has excellent ROS response capability. It has been shown that the polyoxalate bonds in the polymer PVAX are cleaved in an environment rich in hydrogen peroxide, and the drug-loaded nanoparticles can rapidly release CUR. Under the .1 mM H_2_O_2_ condition, the drug release rate of drug-loaded nanoparticles can reach about 90%. *In vitro* and *in vivo* results indicate that these ROS-responsive PVAX-NP can be used as a powerful anticancer drug delivery platform in chemotherapy (Huang et al., 2019).

Jeong et al. reported a thermal gel formed by PEG-PCL-PEG triblock copolymers with oxalate groups in the middle of the polymer. The polymer can form micelles with an average size of 100 nm in water. The aqueous solution of PEG-PCL-PEG triblock copolymer was in the gel state at 37°C (25.0–37.0 wt%). Based on the ROS responsiveness of oxalate groups, the gel formed by triblock copolymers will undergo a gel-sol transition in H_2_O_2_. *In vivo* experiments have demonstrated that the prepared triblock copolymers can form *in situ* gels and carry and release drugs sustainably. PEG-PCL-PEG micellar thermal gels are promising injectable materials with ROS-triggered degradability for biomedical applications (Lee and Jeong, 2019).

## 3 Delivery system based on ROS-responsive polymers

### 3.1 ROS-responsive nanoparticalnanoparticle drug delivery system

Emerging nanotechnologies have resulted in new methods for drug delivery design, known as nanoscale drug delivery systems, that enhance the intelligent and targeted delivery of drugs and nucleic acids to tumor sites (Morachis et al., 2012; Jushan et al., 2021; Ndebele et al., 2021; Liu et al., 2022). Nanomedicine can significantly improve the pharmacokinetics and targeted delivery of anticancer drugs. One of the essential strategies for nanomedicine delivery to tumor sites is to exploit pathological changes in the tumor microenvironment and allow the design to respond to exogenous or endogenous stimuli such as light, temperature, pH, or high enzyme levels (Haitong et al., 2020). The high levels of intracellular ROS in cancer cells have become cancer-specific stimulants that can be used for anticancer therapy; therefore, various ROS-responsive materials are also widely used for tumor delivery with drugs. Hoang et al. synthesized a ROS-responsive poly (ethylene glycol) -poly (methionine) [PEG-P (Met)] that enabled the safe and effective delivery of a pro-oxidant drug piperolamine (PL), to cancer cells. Our results indicate that ROS-responsive PEG-P (Met) micelles are safe and effective delivery vehicles for intracellular drug PL delivery and can provide selective pro-oxidative therapy for cancer (Hoang et al., 2021).

ROS-sensitive approaches are more tumor-specific and thus promise to enhance cancer cell exposure to therapeutic molecules. Xu et al. reported an innovative ROS-responsive polyprodrug. The new NP platform consists of the following key components: A ROS-responsive mitoxantrone (MTO) multiprogenitor that self-assembles with lipid polyethylene glycol (lipid-PEG), then to form nanoparticles by encapsulated in near-infrared (NIR) nanoparticles. In addition, to overcome the barrier of poor penetration of NPs into tumor tissue, these NPs are modified with internalized RGD peptides (iRGD). Both *in vivo* and *in vitro* studies have shown that this nano-platform significantly inhibits tumor cell growth (Xu et al., 2017).

**FIGURE 1:**
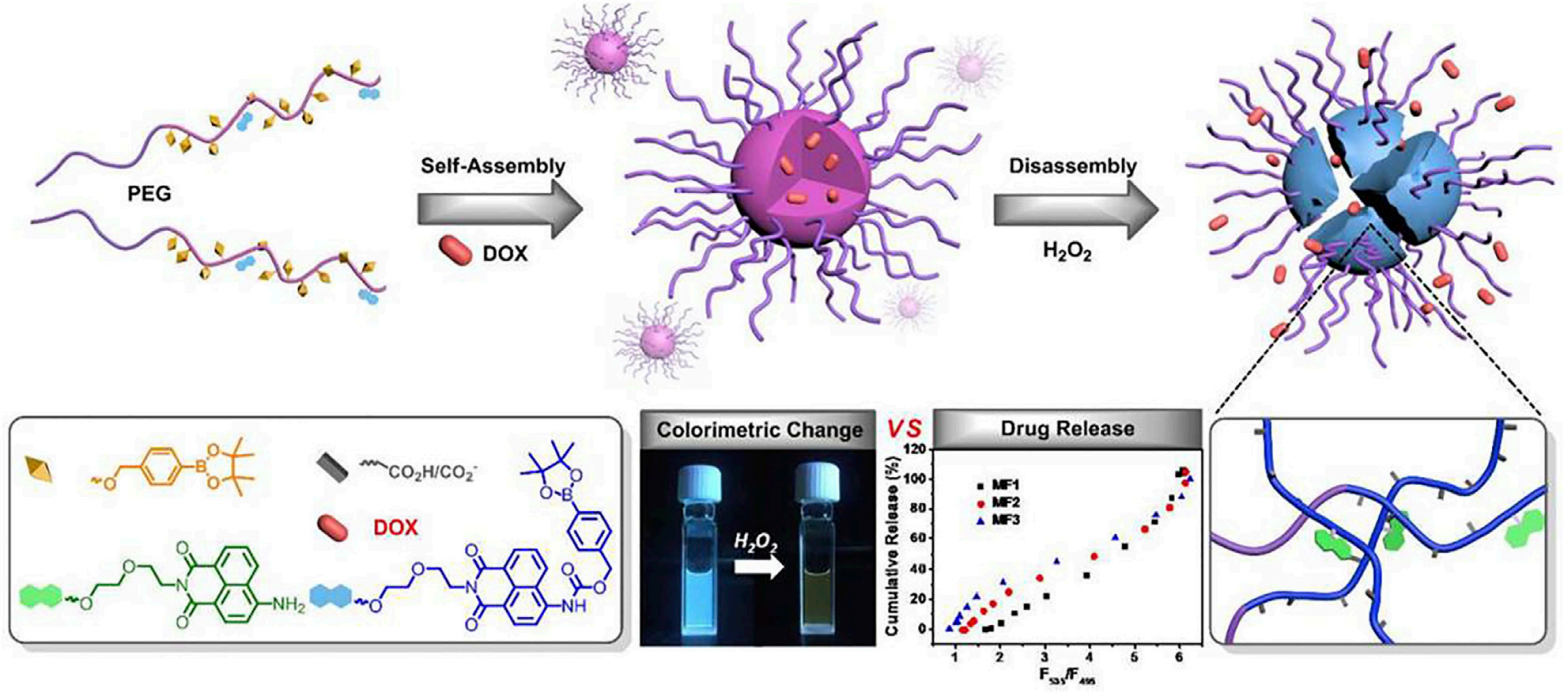
Schematic illustration of a kind of ROS-responsive NPs delivery system. Reproduced from Zhang et al. (2018), with permission from American Chemical Society.

ROS stress is associated with pathologic diseases such as inflammatory diseases and cancer. Zhang et al. reported a ROS-responsive therapeutic drug-polymer nanoparticle that fluorescently self-reports the release of *in vitro* or intracellular drugs under a ROS trigger (Schematic illustration showed in Figure 1). Fluorescent nanoparticles are formed from amphiphilic block copolymers. Based on the fluorescence monomer of 8-naphthalene dimethylimide, the copolymers with different hydrophobic block lengths can be synthesized by atom transfer radical polymerization of acrylic monomer containing phenyl borate, which can be activated by ROS using PEG-Br as the macromolecular initiator. The copolymer can be stably loaded with hydrophobic drugs. The polymers are triggered to degrade in H_2_O_2_, and the degradation rate depends on the hydrophobic block length and H_2_O2 concentration. Finally, the release of the drug was detected by proportional fluorescence (Zhang et al., 2018).

Positive feedback strategies have also been used in the design of ROS-responsive nanomedicines. Hu et al. reported that a ROS-triggered self-accelerating drug release nanosystem defined as T/D@RSMSNs based on mesoporous silica nanoparticles (MSNs) coated with *ß*-cyclodextrin (β-CD) gating *via* ROS-cleavable thioketone (TK) adaptor could enhance tumor chemotherapy. Anticancer drugs and ROS-producing agents are encapsulated in nanoparticles. Adamantane-conjugated poly (ethylene glycol) chain (AD-PEG) are then anchored by host-guest interaction at the surface. Studies have shown that in human breast cancer (MCF-7) cells, T/D@RSMSNs can not only actively release drugs and ROS producers but also increase the concentration of intracellular ROS, thereby promoting the further release of DOX and enhancing the efficacy of chemotherapy. *In vitro* and *in vivo* experiments also demonstrated that T/D@RSMSNs exhibited more significant antitumor activity in human breast cancer compared with conventional single DOX-loaded ROS-responsive nanocarriers (Hu et al., 2017).

### 3.2 Multi-responsive drug delivery system

Because of the complex microenvironment in pathological tissues, a single stimulus administration strategy does not allow for effective targeted drug use at the site of interest. Dual or multiple stimulus-response delivery systems for different site-specific or spatiotemporal delivery are considered novel and more effective strategies (Morey and Pandit, 2018). Therefore, the development of polymers with multilevel response strategies, such as pH, thermal, enzyme, and photo response, as well as the combination of multiple treatment strategies, such as chemotherapy, radiotherapy, gene therapy, photothermal therapy, and photodynamic therapy, has been the focus of ROS-responsive polymer strategies. Although liposomal nanomedicines are currently limited to clinical trials, we expect that a multi-responsive delivery system will provide new options for drug developers.

**FIGURE 2:**
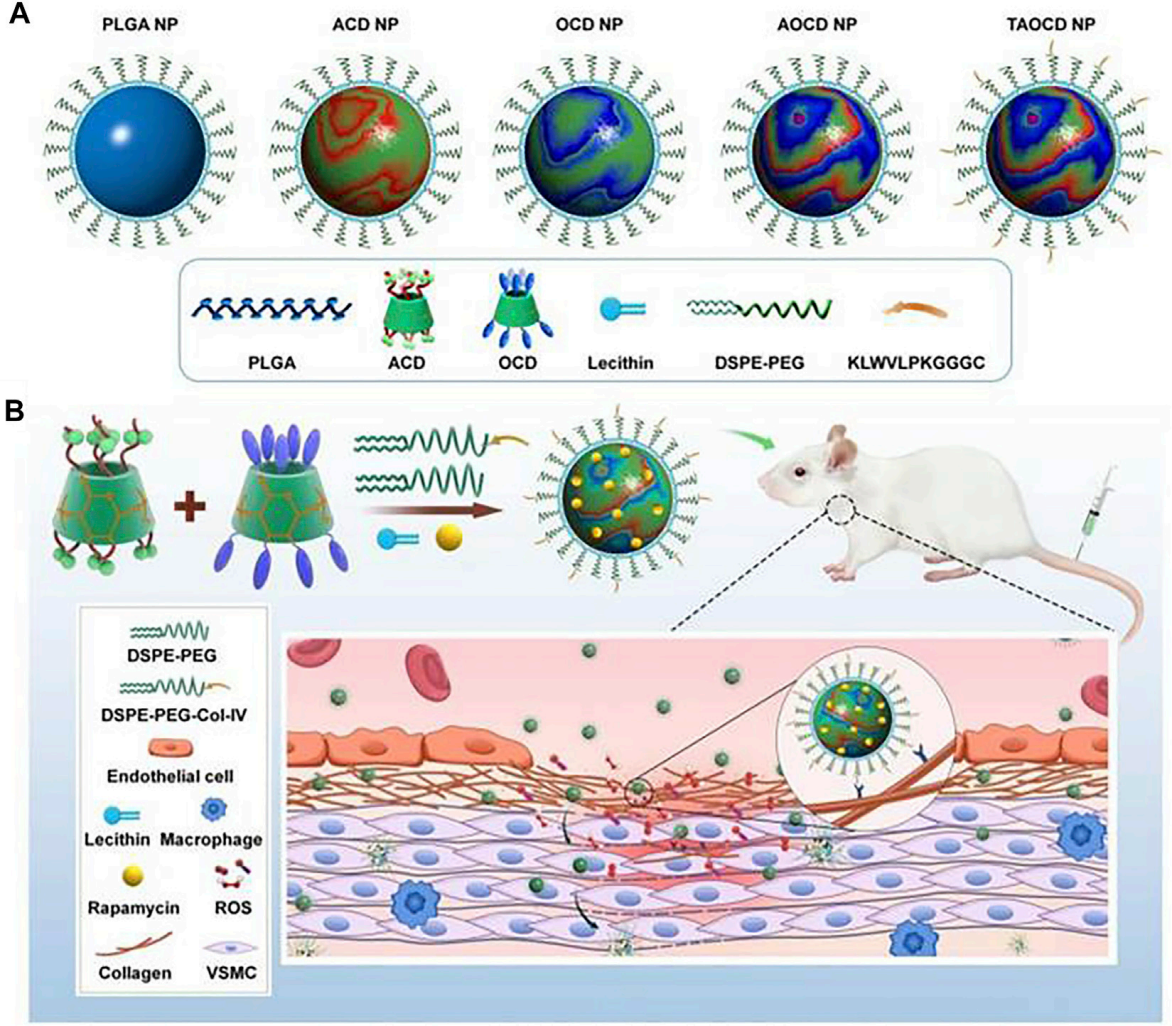
A pH/ROS dual-responsive nanotherapies based on a pH-sensitive *ß*-CD material (ACD) and a ROS-responsive *ß*-CD material (OCD) for targeted treatment of restenosis. **(A)** Schematic illustration of different NPs. **(B)** The targeted treatment of a dual-responsive, targeting rapamycin nanotherapy. Reproduced from Zhang et al. (2018), with permission from Elsevier Ltd.

The pH and ROS dual response nanomedicine is one of the most commonly used multilevel response strategies. Dai et al. (2019), while focusing on the inability of ordinary nanomaterials to release sufficient drugs at tumor sites and the inherent multidrug resistance encapsulated *ß*-lapadone in pH/ROS cascade polymeric prodrug micelles to construct delivery nanosystems with charge reversal capability and self-amplifiable drug release modes. Under the tumor’s weak acidic microenvironment, the micelle systems surface charge will be converted from negative to positive, which can enhance the uptake of tumor cells. It is subsequently dissociated in the ROS environment, releasing *ß*-lapadone and camptothecin (CPT). In addition, the released *ß*-lapadone can further generate ROS and induce self-expansion of micelles and drug release. Both *in vitro* and *in vivo* studies have shown that this synergistic strategy can achieve effective antitumor efficacy. By chemically functionalizing *ß*-cyclodextrin (a cyclic oligosaccharide, *ß*-CD), Zhang et al. combined a pH-sensitive *ß*-CD material (ACD) with an oxidation-sensitive *ß*-CD material (OCD) to construct a pH/ROS dual-sensitive nanoparticle (Figure 2). By adjusting the weight ratio of ACD and OCD, the pH/ROS response-ability can be adjusted to obtain NP with different hydrolysis characteristics in the inflammatory microenvironment. Moreover, rapamycin (RAP) was used as a candidate drug to prepare a nanodrug dual-response delivery system. The dual-response strategy was fully verified using *in vitro* and *in vivo* experiments (Zhang et al., 2020)., Wu et al. built a pH/ROS dual response injectable hydrogel based on the oxidation glucan grafted with benzene boric acid and epsilon-polylysine grafted with caffeic acid. This gel with good biodegradability and self-healing capability can control the spatiotemporal delivery of diclofenac sodium (DS) and mangiferin (MF). *In vitro* and *in vivo* data indicate that the hydrogel is biocompatible and has effective anti-infection, anti-oxidation, and anti-inflammatory effects, thus promoting angiogenesis and accelerating wound repair, which has advantages in the treatment of chronic diabetic wounds (Wu et al., 2022).

### 3.3 ROS-responsive hydrogels

Hydrogels are ideal biomaterials because of their unique morphology and structure. Hydrogels possess unique network structures, excellent hydrophilicity and biocompatibility, and soft physical properties, making them effective materials for various applications. The stimulation response hydrogel is particularly prominent because it can control the material characteristics according to the needs of the tissue and the treatment site to treat various diseases more effectively and improve the methods of tissue engineering and wound healing (Koetting et al., 2015).

**FIGURE 3:**
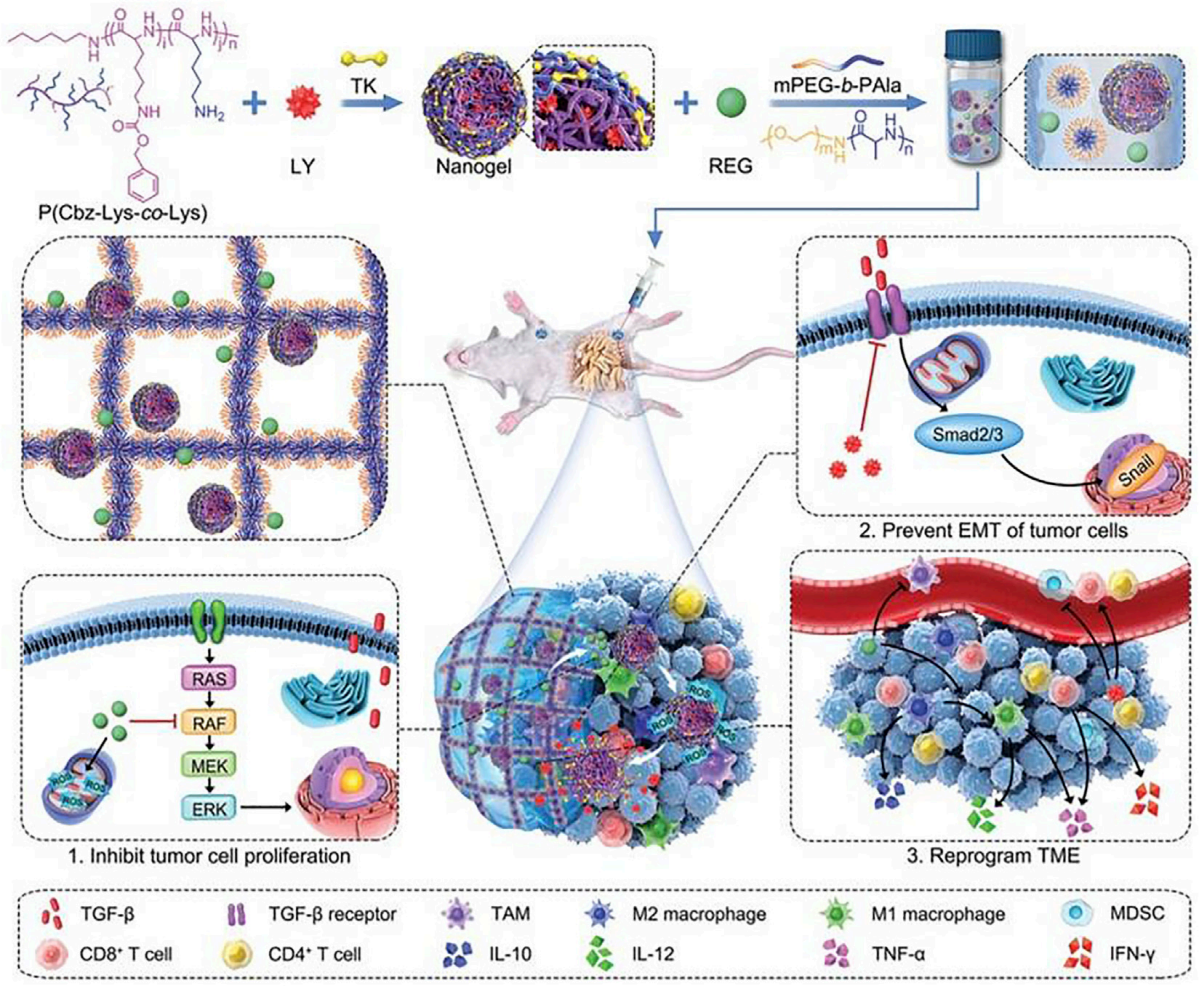
Schematic illustration of preparation and *in vivo* antitumor effect of Gel/(REG + NG/LY). Reproduced from Li et al. (2022), with permission from Wiley-VCH Verlag GmbH & Co. KGaA, Weinheim.

Li et al. (2022) developed a composite of heat-sensitive hydrogels and ROS-responsive nanogels adapted to the tumor microenvironment for precise sequential drug release to enhance molecularly-targeted therapy and amplify immune activation for controlled drug delivery in combination therapy for malignancies (as shown in Figure 3). In the work, a selective transforming growth factor inhibitor was encapsulated in ROS-responsive nanogels and uniformly dispersed with regorafenib (REG) in heat-sensitive hydrogels. After orthotopic injection, drugs and inhibitors can be sequentially released, and studies in cancer treatment models show that this strategy is effective and can potentially improve the prognosis of patients with advanced cancer.

Ruan et al. designed a biological response Gel library by first loading aPD1 into pH-sensitive calcium carbonate nanoparticles (CaCO_3_ (NPs) and then encapsulating it in ROS-responsive hydrogels along with Zebularine (Zeb). This reservoir can respond to acidic pH and ROS in the tumor microenvironment (TME) for code delivery of aPD1 and the hypomethylating agent (HMA), Zeb. Studies have shown that this combination therapy improves the immunogenicity of cancer cells and reverses the immunosuppressive TME, thereby helping to suppress tumor growth and prolonging survival in melanoma mice (Ruan et al., 2019).

Zhao et al. (2020) developed a hydrogel to remove ROS produced by wounds or bacterial infections. The hydrogel was constructed by cross-linking polyvinyl alcohol (PVA) using ROS-responsive connectors. Studies have shown that hydrogels can effectively promote wound closure by reducing ROS levels and upregulating M2-phenotype macrophages around the wound. In addition, this ROS-scavenging hydrogel can be loaded with bactericidal drugs to effectively kill bacteria and release granulocyte-macrophage colony-stimulating factor (GM-CSF) to accelerate wound closure in response to endogenous ROS present in the wound microenvironment. This ROS-scavenging hydrogel can effectively treat various wounds and is a promising strategy for healing complex wounds.

## 4 Conclusion and perspectives

Recently, there has been increased interest in developing responsive polymer drug delivery systems. As one of the triggering drug release mechanisms of reactive polymer drug delivery systems, ROS-responsive materials have become the focus of numerous studies. ROS tends to be overexpressed in many disease sites with high concentrations. The difference between normal and pathological tissues enables ROS-responsive delivery systems to have great potential in many disease applications. The ROS polymer drug carrier can effectively protect the enclosed drugs. This overcomes the water insolubility seen with hydrophobic drugs, quick decomposition seen with protein drugs, and prolongs circulation time while hindering off-target effects. This mode of delivery increases the drug concentration at cancerous tumor sites. Moreover, the application of ROS-responsive materials in myocardial repair, stoke, neuron repair, Parkinson’s disease, and oxidative stress damage repair diseases also demonstrate the potential for therapeutic applications.

The integration of diagnosis and treatment based on ROS-responsive materials is also the current trend. ROS-responsive fluorescent probes can indicate the presence or concentration of the specific ROS. Moreover, the fluorescent probes can be targeted to membrane with specific receptors, cells or specific subcellullar organelles with opposite charges, by modifying specific targeting groups on such probes or making the probes carry charges, thus achieving the spatial and temporal distribution fluorescence imaging of ROS in disease cells or pathogens. Fluorescent diagnostic drugs can also be constructed by linking fluorescent probes and drug molecules together through ROS responsive chemical structures. This kind of fluorescent probe can activate the fluorescence signal and release drug molecules at the same time under the trigger of ROS at the focal site, realizing the integration of disease diagnosis and treatment.

Various ROS-responsive polymer materials and fluorescent probes have been developed, and the ROS responsiveness of related materials can be triggered at low concentrations of pathological ROS to achieve rapid and effective drug release. However, most research on drug carriers is still preliminary, and more detailed research needs to be carried out. Most current delivery systems are not clinically viable; therefore, much work still needs to be done. First, the regulation of ROS responsiveness of the vector itself needs to be improved. ROS is a vital signal transmission medium in the human body. Premature, delayed, or incomplete release of drugs will seriously affect their therapeutic effect. In recent years, there has been increased attention on the specificity and intelligence of polymer nanomaterials. However, these systems are limited by the insufficient EPR effect targeting ability; therefore, more targeting mechanisms, such as the addition of active target groups, should be introduced into the nanomaterials carrier.
